# Supplementary figures and images for: Tensile force-induced cytoskeletal remodeling: Mechanics before chemistry
Source: PLoS Comput Biol. 2020 Jun 10;16(6):e1007693. doi: 10.1371/journal.pcbi.1007693 (PMC7326277; doi:10.1371/journal.pcbi.1007693)

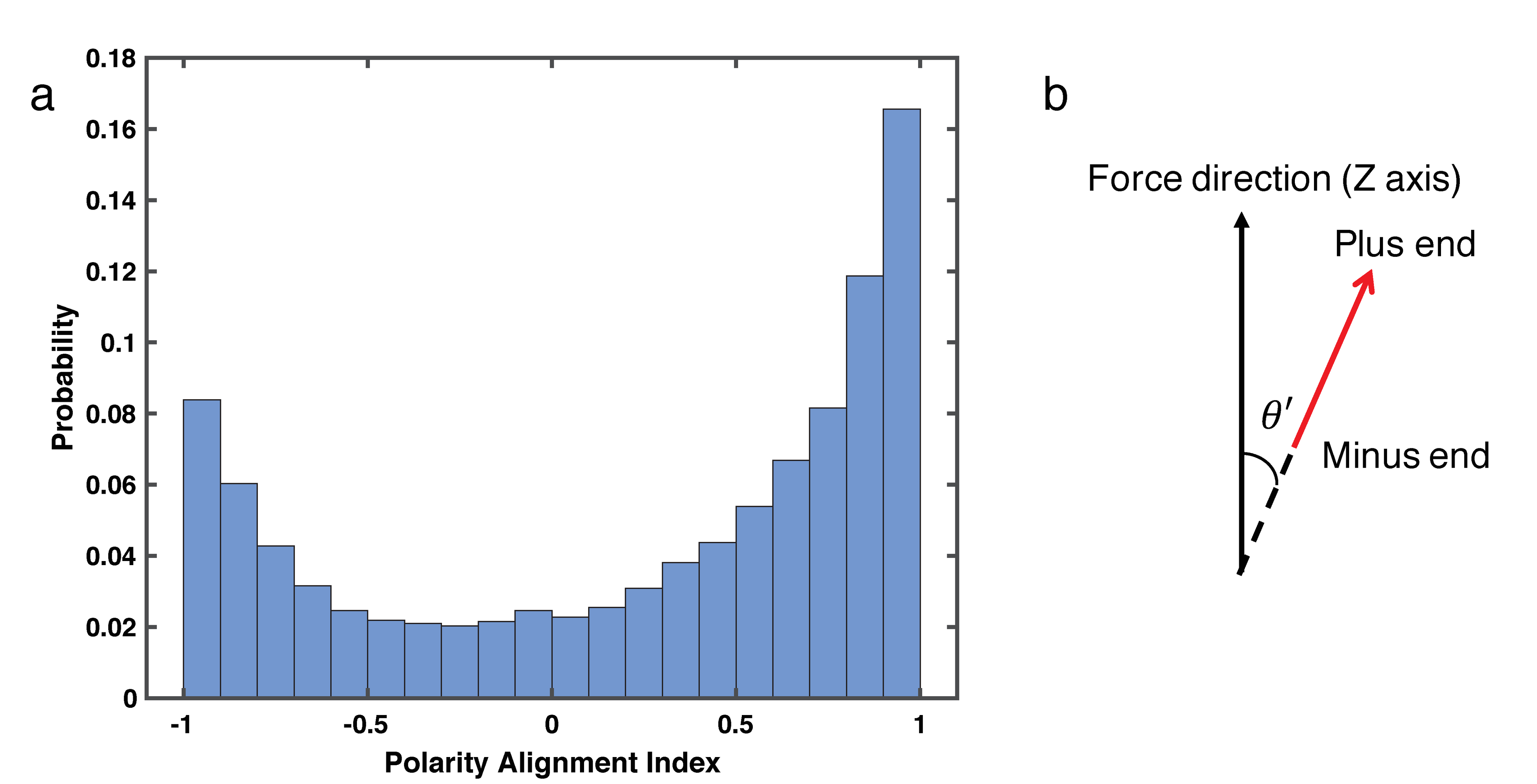

Supplement: S1 Fig — (a) The probability distribution of filament polarity alignment index for bundle-like networks under pulling condition Case i. Data are taken from t = 751s–900s out of 5 duplicated trajectories. (b) The polarity alignment index is defined as cos′θ, where θ′ is the angle between a filament vector and the force direction. The filament vector (red arrow) in this case, considers the polarity of plus end and minus end. (a-b) The distribution spreads across [–1,1], suggesting that the generated actin bundles have mixed polarity. (TIF) [file pcbi.1007693.s001.tif]

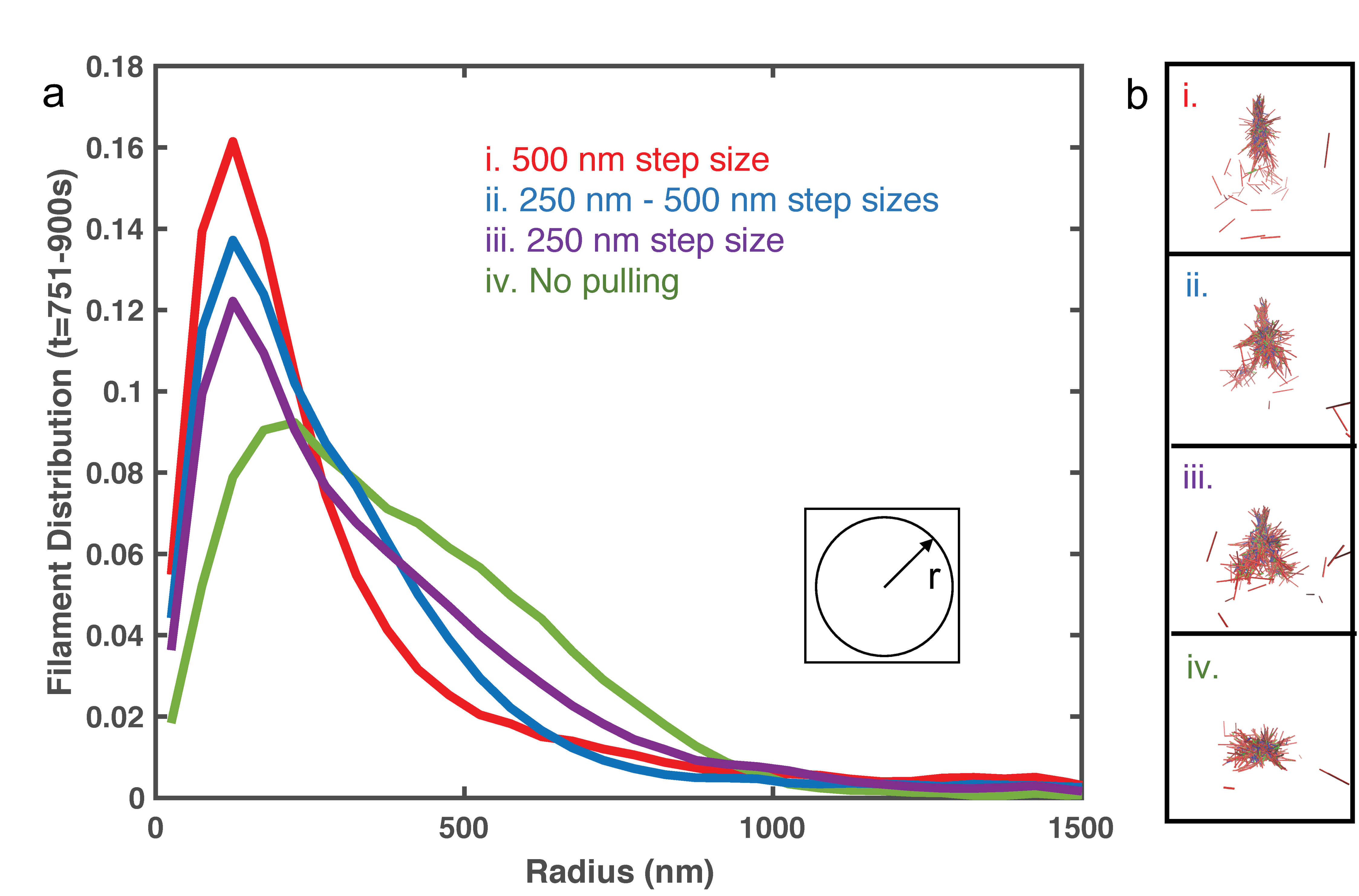

Supplement: S2 Fig — (a) F-actin radial distribution after the last pulling event (t = 751 - 900s) under pulling condition Case i-iv. (b) Representative snapshots at t = 900s for each case. (TIF) [file pcbi.1007693.s002.tif]

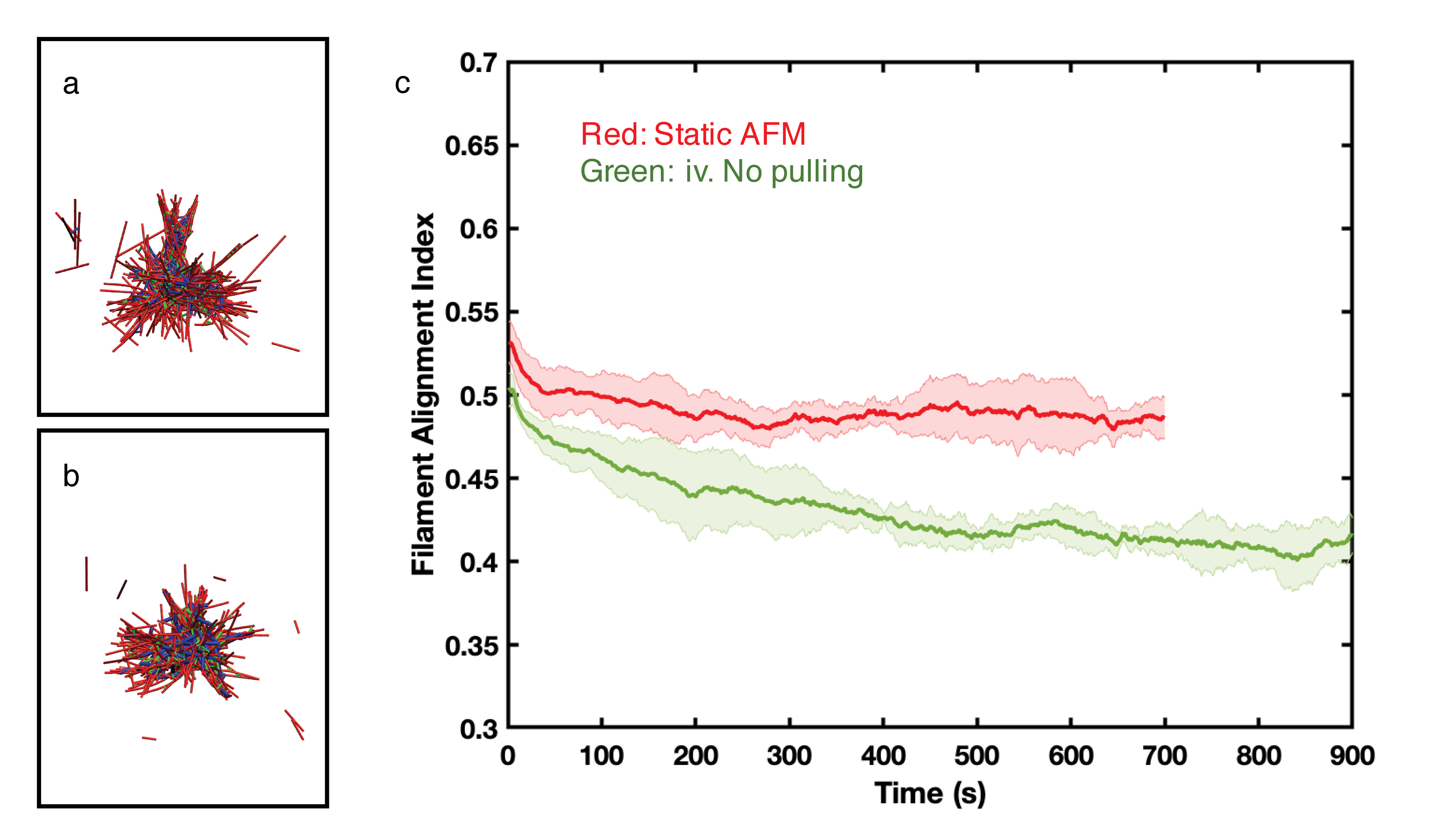

Supplement: S3 Fig — (a) Representative snapshot of actin network with a static AFM probe at t = 700 s. The height of AFM probe is fixed at 1750 nm. (b) Representative snapshot of actin network with no pulling force (control case iv). (c) The alignment index for static AFM-probe (red) and no force condition (green). Error bars represent the standard deviation from the mean from 5 replicate simulations. (TIF) [file pcbi.1007693.s003.tif]

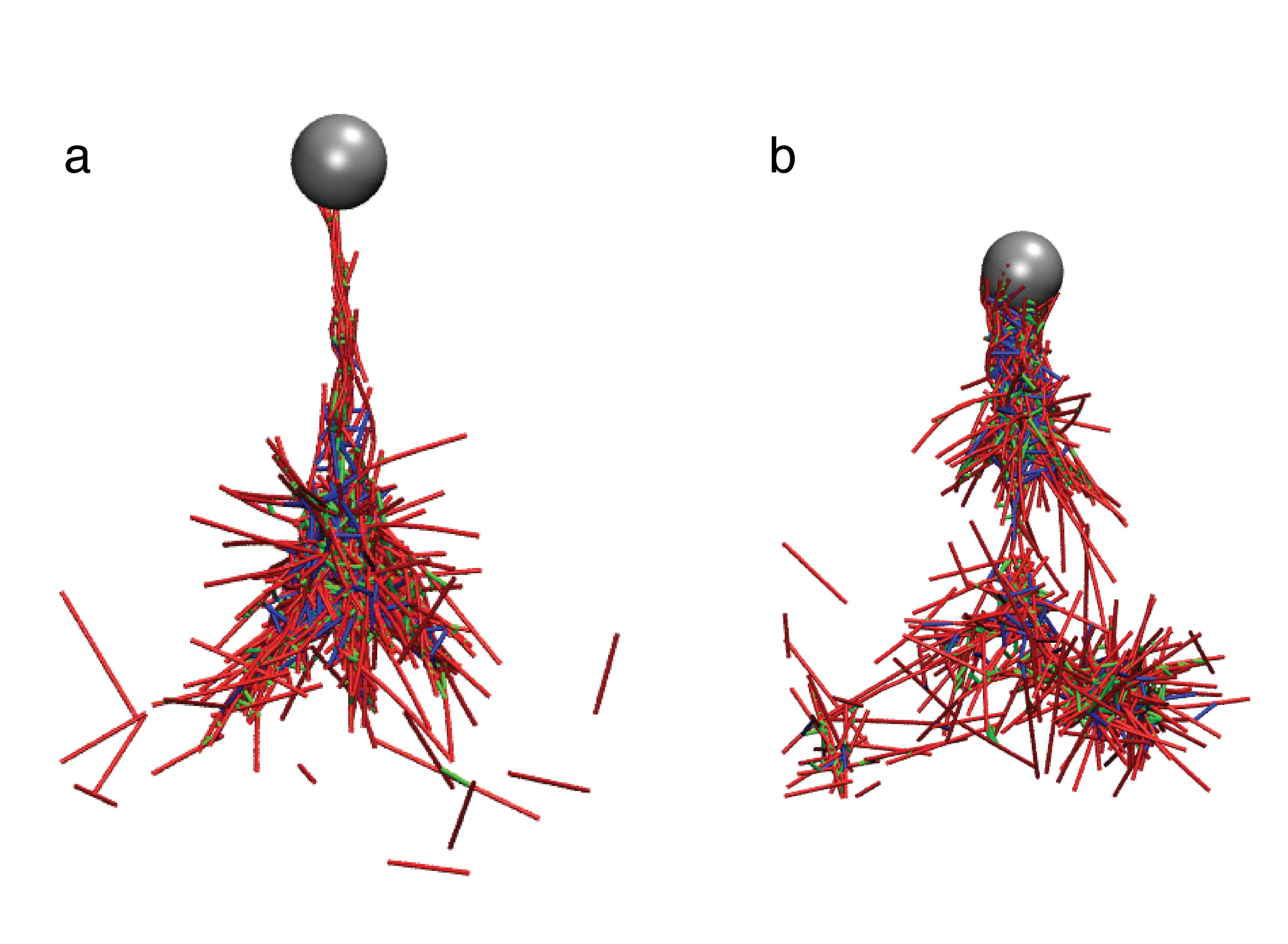

Supplement: S4 Fig — (a) Representative snapshot of actin network with 5 filaments attached to the AFM probe, after the fifth pulling event (d = 500 nm). (b) Representative snapshot of actin network with 60 filaments attached to the AFM probe, after the fourth pulling event (d = 500 nm). Actin filaments, myosin motors, and crosslinkers are shown in red, blue, and green cylinders, respectively. The gray sphere represents the AFM probe. (TIF) [file pcbi.1007693.s004.tif]
