# Supplementary material for: Tensile force-induced cytoskeletal remodeling: Mechanics before chemistry
Source: PLoS Comput Biol. 2020 Jun 10;16(6):e1007693. doi: 10.1371/journal.pcbi.1007693 (PMC7326277; doi:10.1371/journal.pcbi.1007693)
Supplement: Attachment — Submitted filename: ExternalForce_Rebuttal_Final.docx [file pcbi.1007693.s012.docx]

Reviewer #1: This manuscript presents important and interesting findings from molecular simulations and experiments about dynamic remodeling of actin network under the influence of external force. Both experiments and simulations demonstrated fast actin alignment to external force followed by slow myosin-mediated stabilization of the bundled actin network, termed as “mechanics before chemistry” by the authors. The videos of the simulated dynamic remodeling of actin networks under various conditions are particularly enlightening. Findings from this work has strong implications for mechano-transduction and stress fiber formation in cells. The paper is clearly written.

We thank the reviewers for insightful comments. We have revised the manuscript and added new simulations according to the suggestions, as described below.

1.Comparing the default case and the case with 10 s between pulling, it seems like a proper balance between the pulling rate and the biochemical relaxation rate is important for maintaining a continuous actin bundle. Is dynamic pulling necessary though? Could the actin network remodeling be induced just by geometry? For example, if the AFM probe is initially placed 2500 nm away from the initial random actin network and stay there, would an actin bundle similar to that in case iv form?

For VSMCs that reside in a dynamical mechanical environment in vivo, the most relevant assumption is that external forces are dynamic. That is why our experiment and modeling efforts focused on dynamical pulling.

In the experiment, if the probe does not touch the cell, there is no change in actin remodeling. Since large pulls are not physiological, we have not tried to apply a large static pull on the cell. However, based on our experience we can assume that: if the cell has formed strong, mature focal adhesions with the substrate, the probe would either detach from the cell if its adhesion to the cell is weak comparatively with the pulling force applied, or locally rip the cell membrane off if the probe adhesion is strong; if the cell adhesion to the substrate is weak, the probe would eventually lift the cell off the substrate.

In the simulation, to examine whether the dynamic pulling is necessary, we further run new simulations with a static AFM probe pulling. Depending on the number of actin filaments initially attached to the probe, the results varied slightly. But in general, the network behaved similarly to the control case, they form a globular cluster and not bundles. The result is added to Results Section.

In other cases, evidence has shown that dynamic pulling is not necessary for fiber bundling, as was shown in an in vitro assay of actin fiber assembly (e.g., Thoresen et al. Biophys J. 2013). However, this type of experiment is beyond the scope of this work. We have addressed this point in Discussion.

2.Seems like Case iv without pulling was simulated without the 30 AFM-attached actin filaments. Addition of the AFM-attached filaments alone (without pulling) should induce actin alignment, which explains why in Fig.3c Case i-iii has slightly higher alignment index than case iv without pulling in the first 150 seconds. This effect is very weak though, probably because the AFM probe is initially right on top of the initial random actin network. Related to the question above, if the AFM probe is initially set farther away from the initial actin network, would the above effect become stronger?

The interpretation is correct. The slightly higher alignment index for Case i-iii is due to the 30 AFM-attached actin filaments, which are almost parallel to the Z-axis in the beginning of the simulation. However, setting the AFM probe farther away from the initial actin network has very little impact on the alignment index. For example, by setting up AFM probe 500nm above the current position, the mean alignment index is 0.53 (5 replicas), almost the same as the Case i-iii.

3.“With a value between 0 and 1, the alignment index equals to 1 for perfect alignment with the Z-axis, 0.5 for completely random, and 0 for alignment perpendicular to the Z-axis.” According to the definition of the alignment index, average of cos(theta), completely random directions of actin between 0 and pi/2 should give an alignment index of 2/pi = 0.64. Why would it be 0.5 in this case? It is also noted that in Fig. 3c, case iv, the alignment index initializes around 0.5 and gradually drops to around 0.4. Why would the index drop to 0.4 for globular actin cluster?

We thank the reviewer for pointing out the typo in equation 1, where Delta-Z was missing from the denominator. The correct definition of alignment index would indeed equal to 1 for perfect alignment and 0.5 for complete orientational randomness. In the simulation, filaments are initialized by randomly generating coordinates in x, y, and z directions. The mean alignment index of such a random network is around 0.5 at t= 0. The decrease of alignment index in the globular cluster (control case iv) is due to the boundary effect of the simulation. In the control case without pulling, we displace the upper boundary in the same way as in the AFM-probe pulling Case i, while the actin globular cluster remains near the bottom of the elongated simulation box. The lower boundary of the simulation box flattens the globular cluster slightly (Fig3b shows that the globular cluster is flatter at its bottom than its top). This alignment change in the control case can be considered as a systematic error in the simulation, which is much smaller in the pulling cases when the actin bundles move away from the bottom of the simulation box.

Reviewer #2: In their manuscript, Li and Ni et al. investigate a fundamental question in the cellular mechanotransduction field: how do cells sense and react to externally applied mechanical stimuli? To do this, they combine experiments performed in living cells with computational modeling performed in silico to investigate the ability of the actin cytoskeleton within a cell to dynamically respond to tensile external force. Specifically, their experiments consisted of imaging and quantifying the reorganization of the actin cytoskeleton within living vascular smooth muscle cells (VSMCs) expressing mRFP1-tagged actin as an external tensile force was applied to their apical plasma membrane via a fibronectin-functionalized atomic force microscopy (AFM) probe. They then used computational modeling to simulate the mechanical structural adaptation of active actin cytoskeleton networks in response to externally applied tensile force in a manner similar to their AFM-based VSMC experiments. Based on the results of their experiments and modeling, the authors conclude that mechanical structural adaptation occurs before chemical adaptation during actin bundle formation. Basically, Li and Ni propose that actin filaments first align in the direction of the externally applied tensile force, which results in their anisotropic orientation followed by the chemical evolution of the actin network into a dense bundle-like geometry. They call this a “mechanics before chemistry” model of actin cytoskeleton remodeling in response to externally applied force. While I feel that this manuscript tackles an important and timely question in the field of cellular mechanobiology, I have several major and minor issues that I strongly feel that the authors need to address before I can recommend this work for publication at PLoS Computational Biology. In addition, there were several grammatical/writing issues that I would like the authors to address. These issues are all described below.

We thank the reviewer for extremely constructive comments. All responses are listed under the comments as below.

Major Issues:

1)The authors need to do a better job of motivating their work. Why is it important that they “ask how tensile force induces cytoskeletal remodeling and the active formation of actin bundles” in VSMCs?

We edited the introduction to address the motivation. Axial stress in the vessel wall arise from longitudinal tethering of the arteries to surrounding connective tissue (Lawrence and Gooch). Even though axial stress (i.e., tensile force) has been known as an important mechanical stressor, less attention was given to study its biomechanical effects at cellular level. In brief, we observed that stress fibers directional alignment and actin intensity increase after applying tensile force to VSMCs. Inspired by these observations, we aimed to understand how actin remodeling contributes to the cellular adaptation under tensile force.

2)Where is the AFM probe placed on the apical surface of the VSMCs? Is it placed in the same relative position? Is it place on or away from the nucleus? More information is needed.

Based on the literature and our own experience, location of the AFM measurements matters (e.g., Docheva et al., J Cell Mol Med 12 (2008) 537-552). We do all our measurements or stimulation experiments by placing the AFM problem at the same relative position on the apical surface of the VSMCs, midway between the edge of the nucleus and the edge of the cell. We have clarified this point in the revised paper.

3)The authors need to explain why they chose the specific concentrations of the molecules included in their computational models. What are the references that these concentrations were pulled from and in which cell types?

Myosin-II concentration is about 2 µM in Dictyostelium discoideum (Robinson et al. BMC Cell Biol. 2002. Egelhoff et al. Cell 1993) and alpha-actinin concentration is found to be around 2 µM in Dicty as well (Pollard, 2000). We use a concentration of 20 µM for actin, which is consistent with the physiological range of 24 µM reported in many papers (e.g., Xu and Wirtz, JBC 2000, Tseng and Weitz, Biophys J 2001). The concentrations of actin, motors and crosslinkers in the computational model are also used in prior computational modeling works (Popov, PLoS CB, 2016). These explanations and references were added to the method section.

4)Why were only 30 filaments attached to the AFM tip in the simulations shown in Fig. 2? What happens if the number of attached filaments is increased or decreased? Are the filaments always attached to the AFM tip or do the have a dissociation constant?

We choose 30 filaments based on the number of filaments in an actin bundle to be 10-30 (Tojkander, JCS, 2012). With too little filaments attached to the AFM tip, the free filament pool and the AFM probe would disconnect as the probe is moving up. On the other hand, increasing the number of attached filaments may hinder the network connectivity. By keeping the total number of filaments and total actin concentration the same, increasing the number of AFM attached filaments would reduce the number of free filaments. When the filament density in the free pool becomes too low to generate global contractility, some free filaments are unable to connect to the actin bundle. These filaments are always attached to the AFM probe and do not dissociate. If filaments dissociate from the AFM tip, bundles would tend to contract into more cluster-like geometry. We add these explanations to Results.

5)The simulation described in Fig. 2A does not seem to be physiologically relevant to the AFM experiments performed on live VSMCs shown in Fig. 1. I say this because the authors are measuring the axial displacement of fluorescently labeled pre-assembled actin stress fibers caused by the pulling of an AFM tip on the apical surface of a VSMC, while the simulations are pulling on an active network of actin filaments that evolves over the time that tensile force is applied to the network. In addition, the geometry of the stress fibers in the VSMCs is quite noticeably different from the geometry of the simulated AFM experiments. Were the authors to have measured the accumulation of an active cytoskeletal network underneath the adhered AFM as it was pulled away from the apical VSMC plasma membrane, the simulations presented in this work would be more directly comparable.

We would like to clarify that in the experiments, the actin filaments were not only moving in space, but also actively (de)polymerizing and forming bundles, as evident in Video 2 and Figure 6 (Trache, JBO, 2009). The figure below (Lim et al., 2012) exemplifies that in the experiment, a matrix functionalized AFM probe will induce active focal adhesion proteins and actin accumulation under the bead. Actin (red), vinculin (green), and integrin-b1 (red) are recruited by the fibronectin (FN) coated bead (green) to form a strong FA (see arrows). The overlay shows the integrin beta1 ring formed around the FN-coated probe. The AFM probe above the cell is shown by white dashed lines. However, due to imaging resolution constraints we are not able to consistently visualize and quantify the actin changes under the bead.

In the simulations, we focus on tensile force induced actin remodeling, which is a small volume representation of the whole cell response. So, our simulations correspond well to the experiments.

6) I cannot agree with the authors’ description of their simulated active actin networks under tension as being stress fiber-like. While the simulated networks and stress fibers both have mixed filament polarity, the architecture of these two actin structures are quite different. The architecture of a stress fiber is reminiscent of a contractile sarcomere in muscle, which are “blocks of actin filaments of alternating polarity and bands of interdigitating non-muscle myosin” (Pellegrin and Mellor, 2007 J Cell Science). Pellegrin and Mellor go on to say, “the structure (i.e. sarcomere /stress fiber) is held together by α-actinin and also crosslinked by non-muscle myosin”. In contrast, the authors’ simulated active actin networks more closely resemble the actin “comet tails” responsible for the intracellular movement of endosomes, some bacteria (i.e. Listeria monocytogenes and Rockettsia conorii), and some viruses (i.e. Vaccinia and Baculoviruses) (Welch and Way, 2013 Cell Host Microbe; Fehrenbacher et al., 2003 J Exp Biol). Given this disconnect between the VSMC experiments and the simulations, I find it difficult to see how the in silico modeling complements or illuminates the in vitro cellular experiments presented in this work.

We agree that the actin bundles generated in our simulation should not be called stress fibers. We change the term "stress fiber" to "actin bundle" throughout the manuscript. In our simulation, filament length quickly reaches steady state, and the assembly of actin bundle is primary driven by actomyosin apparatus, and the AFM probe displacement. Our simulations add a potential explanation (in addition to filament polymerization) to actin bundle-thickening under tensile stress as shown by increasing in actin intensity after AFM pulling in VSMC experiments. Free filaments in the cell can be recruited to actin bundles. Our simulations suggest that the application of tensile force can potentially induce directional alignments of these filaments, followed by slower thickening of actin bundles. We have added these points to Discussion.

7) I would very much like to see movies of the AFM experiments performed on VSMCs expressing the so-called actin-mRFP1 construct (see Minor Issue #5 below).

The movie is added as Supporting Materials Video 1.

8)In lines 5-6 of the 2nd paragraph of the “Two-step development of actin bundles relies on both faster mechanical alignment and slower chemical stabilization” section of the Results, the authors state, “The control case without external pulling (iv, green line) shows the biochemically driven F-actin accumulation, as a result of myosin-induced contractility”. Why do they authors think that this is all due to myosin-induced contractility and not also due to alpha-actinin-induced bundling and/or actin polymerization?

We thank the reviewer for pointing out the inaccurate statement. The contraction of the network is indeed induced by actomyosin apparatus, which consists of myosin motors, alpha-actinin, and actin filaments. We edited the sentence to “The control case without external pulling (iv, green line) shows the biochemically driven F-actin accumulation as a result of actomyosin apparatus activities.”

9)Why do the authors only include “4 possible binding sites for myosin motors and crosslinkers” for a 40 subunit 108 nm long cylinder of an actin filament? This is a gross underestimation, as a myosin-II motor can interact with each actin subunit in a filament. Therefore, I would assume that there were 40 myosin-binding sites per actin filament cylinder. What happens if the number of binding sites were increased in the computational models?

Choosing to model motor binding using the discretized binding site assumption is mainly an effort to improve computational efficiency. In our computational model, myosin motor is implicitly represented as a single potential acting on two neighboring cylinders. Considering the width of a non-muscle myosin II mini filament is around 20 nm (Pollard, JCB, 1982), we choose binding sites every 10 subunits (27 nm). Although we sacrifice some precisions in this assumption, the system will rescale its reaction rates for motor binding and motor walking based on the number of binding sites. For example, in this work, myosin walking speed under zero force is modeled as

$$k_{walk}^{0}=\frac{(1-\rho)}{\rho}k_{bind}=10.8 nm/s,$$

where $\rho=0.1$ is duty ratio, and $k_{bind}=0.2 s^{-1}$ is the binding rate of a single motor head. Under the 4-binding site assumption, the myosin walking speed is 0.4 binding sites per second, and the space between two binding sites is 27nm. If we increase the number of binding sites to 16, then the speed will be adjusted to 1.6 binding sites per second, and the space between two binding sites become 6.75nm.

10)I would recommend that the authors tone down their conclusion that their “mechanics before chemistry” hypothesis is novel. It is well known in mechanobiology that the timescales of mechanotransduction range from milliseconds to seconds for the stretching of mechanosensor proteins to the polymerization of actin, respectively.

We have removed the word 'novel' from the statements. We agree that the timescale for mechanically induced protein configuration changes are much shorter than that for polymerization of actin. However, the timescale for actin filaments /bundle alignment is different from protein conformation change, and the separation of timescale between the mechanical and chemical responses has not been demonstrated in actin remodeling under external stress. Our work integrated experimental and modeling studies to clearly demonstrate this time separated two-step process.

Minor Issues:

1)In the legend of Fig. 2, the authors need to state how long the 300 free actin filaments are that are in their simulations.

We have added "At the beginning of simulations, all filaments are 0.108 μm long (40 actin subunits). The input G-actin concentration is much higher than the equilibrium concentration, making filaments rapidly elongate. An average length of 0.8 μm is achieved and maintained after around 40s of simulation."

2)In the 5th line of the 4th paragraph of the Discussion, the authors might elaborate on which specific signaling pathways might be triggered by stretching focal adhesion proteins.

Mechanical stretch alters conformation of integrins (Katsumi et al, JBC 2005) such that their cytoplasmic β-tails provide binding sites for c-Src and FAK (Katsumi 2005) (Fig. 1), further induce assembly of an adhesion complex at the site of force application (Alenghat and Ingber, 2002; Geiger. Curr Opin Cell Bio 2001). This process is followed by actin stress fiber remodeling, which is necessary to redistribute physical forces needed for cell contraction and to enable cell adaptation to the extracellular microenvironment (Chen, PNAS 2013; Zhang, J Physiol 2006). A simplified text was added in the Discussion section.

3)In the last paragraph of the Discussion, the authors state, “we integrated in vitro and in silico modeling”. However, I do not understand what is meant by “in vitro modeling” in this statement. It seems to me that the authors integrated cellular biophysical experiments with in silico modeling instead.

We corrected the sentence to "we integrated *in vitro* cellular biophysical experiments and *in silico* modeling to investigate..."

4)In the last sentence of the Discussion, the authors state, “This result suggests….which can have important implications to mechano-signal transduction. Perhaps they might provide some examples of such “important implications”? This would give the reader a sense of where the authors plan on going next with this line of research.

We changed the sentence to “This study lays the ground work for further investigations related to predicting cellular adaptation to mechanical stimulation for vascular cells presenting increased cellular stiffness as in hypertension and aging.”

5)The authors state that they are expressing an actin-mRFP construct that they received from the late Michael Davidson in VSMCs to visualize the actin cytoskeleton. However, a quick search of Michael’s fluorescent protein construct collection for a red actin construct suitable for mammalian expression revealed that he had a construct named “mRFP1-actin-7”, not one named “actin-mRFP”. Therefore, the authors should change all mentions of “actin-mRFP” to “mRFP1-actin-7”. This change is important for others to be able to reproduce the authors’ results. Moreover, the way that the authors write “actin-mRFP” suggests that the mRFP is fused to the C-terminus of actin, which is not correct.

We correct all “actin-mRFP” to “mRFP1-actin-7”.

6)In the Materials and Methods, the authors cite direct their readers to reference #45 for detailed information regarding the “integrated microscope system” used in this manuscript. While I understand that they do not want to have to go into all of the details of their previously described imaging system, I do feel that they need to at least provide their readers with the following information:

a.The name of the microscope company that the base of the microscope was built on.

b.The numerical aperture value for their 60x objective and the name of the microscope company that manufactured it.

c.The cameras used and the companies that made them.

d.The light source (I assume that they are lasers, but which ones and from where?).

e.The information about the excitation and emission filters used.

f.The software used to drive the imaging system.

We have added all the suggested details to “Vascular smooth muscle cell imaging” section.

7)What was the concentration of fibronectin used to coat the AFM probe?

The AFM probe consists of a bead at the end of the cantilever that was first coated with 1 mg/ml avidin (Sigma, St. Louis, USA) and then cross-linked with biotinylated fibronectin, which was prepared by mixing 1 mg/ml FN (F4759, Sigma) with 10 mg/ml Sulfo-NHS-LC-Biotin (21335, Pierce, Rockford, IL, USA),

8)In the “Simulation Methods” section of the Methods, I do not understand what the authors mean by the phrase “strong axial stretching stiffness”.

We have changed the sentence to “The cylinder itself is unbendable, and the radial deformation of filaments is realized by bending between two neighboring connected cylinders.” to avoid confusion.

9)In the “Simulations Methods” section of the Methods, the authors need a reference for the following statements:

a.“Myosin motors are modeled as harmonic springs…pm the non-muscle myosin II”.

b.“Crosslinking proteins are also modeled as harmonic springs…(30-40 nm)”.

We have added two new references for these two statements.

Writing Suggestions:

1)In the 3rd line of the 2nd paragraph of the Introduction, the authors state, “is critical in understanding”. They should change the “is” to a “for”.

2)In the 6th line of the 4th paragraph of the Introduction, the authors should change “myosin activation” to ”myosin-II activation”.

3)In the 1st line of the 5th paragraph of the Introduction, the authors should remove the “the” following “upon”.

4)In the 3rd line of the 5th paragraph of the Introduction, the authors should insert the word “software” following “Network)”.

5)In the heading of the 1st section of the Results, the authors should change the “of” before “live” to “in”.

6)In the 1st line of the 2nd paragraph of the “Two-step development of actin bundles relies on both faster mechanical alignment and slower chemical stabilization” section of the Results, the authors need to change “filament” to “filaments” and “keeps” to “kept”.

7)In the 3rd line of the 2nd paragraph of the “Two-step development of actin bundles relies on both faster mechanical alignment and slower chemical stabilization” section of the Results, the authors need to change “calculate” to “calculated”.

8)In the 5th line of the 3rd paragraph of the Discussion, the authors should change “isotropically” to “isotropic”.

9)In the 2nd line of the last paragraph of the Discussion, the authors should insert “actin” before “cytoskeleton”.

10)In the last sentence of the Discussion, the authors should change the “to” to a “for”.

11)In the 2nd to last sentence of the “Experimental Methods” section of the Methods, the authors should change the “of” before “Michael” to “from”.

12)In the “AFM mechanical stimulation to VSMCs” section of the Methods, the authors should make the following changes:

a.Change the “to” before “VSMCs” to “of” in the heading of this section.

b.Change the two “consists in” to “consists of”.

13)In the “Three-dimensional image analysis” section of the Methods, the authors should make the following change:

a.In the penultimate sentence before the Alignment Index equation, the word “was” needs to be changed to “were”.

14)In the “Simulations Methods” section of the Methods, the authors need to make the following changes:

a.In the 2nd paragraph, the “allowing” found in the sentence that begins with “Only plus ends of these filaments” needs to be changed to “allowed”.

b.In the 3rd paragraph, the “tips” in the sentence that begins with “Each probe displacement” needs to be changed to “tip” and the “generating” needs to be changed to “applying”.

c.In the 3rd paragraph, the “are” in the sentence that begins with “Since all AFM” needs to be changed to “were”.

15)In the legend of Fig. 1, the authors need to delete the “were” from the description of panel (a).

16)In the legend of Fig. S1, the “is” in the sentence that begins with “The distribution spreads” needs to be changed to “are” and ”a” needs to be inserted before “stress”. In addition, a reference for this statement needs to be provided.

We appreciate the reviewer for the thorough review and have carefully edited the manuscript to incorporate all these suggestions.
